# Attenuation of Red Blood Cell Storage Lesions with Vitamin C

**DOI:** 10.3390/antiox6030055

**Published:** 2017-07-12

**Authors:** Kimberly Sanford, Bernard J. Fisher, Evan Fowler, Alpha A. Fowler, Ramesh Natarajan

**Affiliations:** 1 Department of Pathology, Director, Transfusion Services, Virginia Commonwealth University, Richmond, VA 23298, USA; kimberly.sanford@vcuhealth.org; 2 Department of Internal Medicine, Virginia Commonwealth University, Richmond, VA 23298, USA; bernard.fisher@vcuhealth.org (B.J.F.); evan.fowler@vcuhealth.org (E.F.); alpha.fowler@vcuhealth.org (A.A.F.)

**Keywords:** vitamin C, RBC storage lesions, mean fragility index, β-spectrin, scanning electron microscopy

## Abstract

Stored red blood cells (RBCs) undergo oxidative stress that induces deleterious metabolic, structural, biochemical, and molecular changes collectively referred to as “storage lesions”. We hypothesized that vitamin C (VitC, reduced or oxidized) would reduce red cell storage lesions, thus prolonging their storage duration. Whole-blood-derived, leuko-reduced, SAGM (saline-adenine-glucose-mannitol)-preserved RBC concentrates were equally divided into four pediatric storage bags and the following additions made: (1) saline (saline); (2) 0.3 mmol/L reduced VitC (Lo VitC); (3) 3 mmol/L reduced VitC (Hi VitC); or (4) 0.3 mmol/L oxidized VitC (dehydroascorbic acid, DHA) as final concentrations. Biochemical and rheological parameters were serially assessed at baseline (prior to supplementation) and Days 7, 21, 42, and 56 for RBC VitC concentration, pH, osmotic fragility by mechanical fragility index, and percent hemolysis, LDH release, glutathione depletion, RBC membrane integrity by scanning electron microscopy, and Western blot for β-spectrin. VitC exposure (reduced and oxidized) significantly increased RBC antioxidant status with varying dynamics and produced trends in reduction in osmotic fragility and increases in membrane integrity. Conclusion: VitC partially protects RBC from oxidative changes during storage. Combining VitC with other antioxidants has the potential to improve long-term storage of RBC.

## 1. Introduction

Red blood cell (RBC) transfusion is a life-saving procedure whose primary objective is to sustain tissue and organ oxygenation in case of acute anemia due to massive hemorrhage or chronic anemia secondary to bone marrow dysfunction. RBC units, prepared by plasma removal and leukocyte depletion, are stored for 42 days [1]. This extension of RBC shelf-life is possible due to the combined application of preparation methods, suitable storage additive solutions (CPD—citrate phosphate-dextrose, saline-adenine-glucose-mannitol (SAGM) or Optisol (AS-5)), polyvinyl chloride blood bags, and storage at 4 °C [2]. However, despite these measures, stored RBCs undergo deleterious metabolic, structural, biochemical, and molecular changes collectively referred to as “storage lesions” [3]. Storage lesions are characterized by ATP depletion, loss of 2,3-diphosphoglycerate (2,3-DPG), glutathione (GSH), and nicotinamide adenine dinucleotide (NADH/NADPH) depletion with subsequent oxidation of hemoglobin, exhaustion of the endogenous antioxidants, leakage of lactate, lactate dehydrogenase (LDH), hemoglobin, and potassium ions into the suspending medium. Membrane micro-vesiculation as well as reversible and irreversible morphological changes also occur [4,5,6,7,8].

A major contributor to storage lesions is oxidative stress that progressively increases over the storage period due to consumption of endogenous anti-oxidants. Wither and colleagues identified oxidative modifications of functional residues of the hemoglobin beta chain [9]. Rinalducci and colleagues detected progressive linkage of cytosolic proteins to the cell membrane that included antioxidant and metabolic enzymes. Their detailed analysis of aged RBC membranes suggested that peroxiredoxin-2 could serve as a marker of oxidative stress [10]. Studies using mass spectrometry and electron microscopy have shown that various factors including altered cation homeostasis, reprogrammed energy, and redox metabolism contribute to the progressive accumulation of oxidative stress and storage lesions in stored RBCs [8]. Oxidative stress, in turn, promotes oxidative lesions to proteins (carbonylation, fragmentation, denatured hemoglobin) and lipids (peroxidation), which then contribute to the altered physiology of stored RBCs [8,11,12]. 

Pharmacological concentrations of VitC induces oxidative stress and GSH depletion while promoting increased glucose flux through the oxidative pentose phosphate pathway (PPP) in erythrocytes. Zhang and colleagues showed that erythrocytes incubated with VitC exhibit hemolysis [13]. Hemolysis was intensified in erythrocytes obtained from glucose-6-phosphate dehydrogenase (G6PD) deficient patients. Of importance in this work, erythrocytes were salvaged in Zhang’s work and in reports by others [14] by the addition of antioxidants, by alterations of pH [15] or by addition of alkaline CPD, which preserves erythrocyte 2,3-DPG. [16].

RBC deformability is critical to RBC function, especially in the microvasculature where impaired deformability adversely affects capillary perfusion [17]. Oxidative injury also induces formation of RBC membrane microparticles with release of bioactive lipids that promote pro-inflammatory/pro-coagulant responses in the recipient, resulting in transfusion-related acute lung injury (TRALI) [18]. Another component of storage lesions is increased sub-lethal injury. Sub-lethal injury refers to the damage inflicted upon cells that does not result in their immediate hemolysis and that is exacerbated by oxidative stress [19,20]. It is expressed using the mechanical fragility index (MFI). Higher MFI values correspond to greater amounts of sub-lethal injury.

The addition of antioxidants to stored RBCs has the potential to mitigate oxidative injury and thus storage lesions. Recent studies indicate a renewed interest in the use of pharmacologic doses of vitamin C (VitC) to stored RBCs. Raval et al. showed that VitC exposure significantly reduced mechanical fragility and hemolysis in stored RBCs [19]. Stowell et al. demonstrated that the addition of an ascorbic acid solution to stored murine RBCs improved post-transfusion recovery while decreasing microparticle formation and allo-immunization [21]. Czubak et al. used a combination of sodium ascorbate and trolox to inhibit hemolysis and lipid peroxidation, while enhancing total antioxidant status [22]. These studies indicate that RBC preservation using optimal concentrations of VitC may prevent storage lesions.

We investigated the effects of reduced VitC (ascorbic acid: 0.3 mmol/L, 3 mmol/L) and oxidized VitC (dehydroascorbic acid [DHA], 0.3 mmol/L) on oxidative stress mediated changes in RBC’s stored at 4 °C for 56 days. We hypothesized that VitC would reduce red cell storage lesions by attenuating osmotic fragility, LDH release, and glutathione depletion by maintaining RBC membrane integrity and structure, thus prolonging their storage duration, and improving their viability and function upon transfusion.

## 2. Materials and Methods

### 2.1. Research Involving Human Subjects

No human subjects were used for this study. However, freshly donated whole blood units from 5 de-identified community volunteer donors that meet the specific donor criteria established by the Food and Drug Administration through the Code of Federal Regulations (CFR), guidance documents and the American Association of Blood Banks (AABB) established professional standards for donor selection were purchased from Virginia Blood Services (Richmond, VA, USA). We declare that this study was carried out in accordance with the Declaration of Helsinki. Further, since no human subject information was gathered, approval by an institutional review board was not required.

### 2.2. Preparation and Storage of RBCs

Freshly donated whole blood units from 5 de-identified donors was purchased from Virginia Blood Services (Richmond, VA, USA). Blood was screened and RBC concentrates were prepared by Virginia Blood Services using standardized protocols of plasma removal and leukocyte depletion, followed by the addition of SAGM to RBCs. Each RBC unit was equally divided into four pediatric storage bags (volume of ~75 mL per aliquot) and supplementations for the study were made to each aliquot at the supplier facility as described below using aseptic technique. Prior to supplementation, an initial baseline sample was collected at the blood supplier facility and transported to the laboratory for analysis. RBCs that passed standard screening tests were transported two days later to the Virginia Commonwealth University Transfusion Medicine Center and stored at 4 °C for 56 days. All subsequent collections were made at the Virginia Commonwealth University Transfusion Medicine Center on Days 7, 21, 42 and 56.

### 2.3. Experimental Design and Study Groups

RBCs in pediatric storage bags were treated with one of four additives: (1) normal saline (saline); (2) 0.3 mmol/L reduced VitC (Lo VitC); (3) 3 mmol/L reduced VitC (Hi VitC); or (4) 0.3 mmol/L oxidized VitC (DHA) as final concentrations and gently mixed. The reduced VitC additive was preservative-free buffered ascorbic acid in water (Ascor L500, McGuff Pharmaceuticals, Santa Ana, CA, USA), pH 5.5–7.0 adjusted with sodium bicarbonate and sodium hydroxide. Oxidized VitC (DHA) was procured from Sigma-Aldrich (St. Louis, MO, USA). DHA was dissolved in saline at 60 °C for 30 min. It was filter sterilized and injected into the pediatric bags using a sterile technique.

### 2.4. Determination of RBC Vitamin C Content and pH

For vitamin C quantification, RBCs were pelleted by centrifugation (10,000 *g*, 10 min at 4 °C) and the supernatant plasma saved for pH determination. Pelleted RBCs were washed with cold saline; deproteinized in 200 µL of cold 20% trichloroacetic acid (TCA) followed by the addition of 200 µL of cold 0.2% dithiothreitol (DTT) to prevent oxidation. RBC lysates were vortexed and centrifuged at 10,000 *g* for 10 min at 4 °C. The supernatants were stored at −80 °C for batch vitamin C analysis. Total vitamin C content was assessed using a Tempol-OPDA-based fluorescence end-point assay as previously described [23].

### 2.5. Mechanical Fragility Test and Percent Hemolysis

The mechanical fragility test and percent hemolysis were performed on each sample as described by Raval et al. [19]. Briefly, at each time point, aliquots from each of the treatments were removed, adjusted to a standard hematocrit (Hct) of 40% with Dulbecco’s phosphate buffered saline (DPBS with calcium and magnesium, GE Healthcare Life Sciences, HyClone Laboratories, Logan, UT, USA) and the hemoglobin (Hb) concentration determined spectrophotometrically (ABX Micros 60; Horiba, Ltd., Kyoto, Japan). Two milliliters of each aliquot were then added to each of five tubes (6 mL, 13 × 100 mm Vacuette blood collection tubes, Greiner Bio-One; Monroe, NC, USA), three of which contained five 3.2 mm steel ball bearings (BNMX-2, Type 316 balls; Small Parts, Inc., Miami Lakes, FL, USA) and two of which did not. The tubes with ball bearings were rocked on a rocker platform for 1 h, while the remaining tubes without bearings were not rocked and served as controls to ascertain the initial concentration of free Hb in each aliquot. After rocking, all tubes were centrifuged twice, and the free Hb concentrations in the supernatants determined spectrophotometrically by light absorbance at 540 nm (Bio-Rad SmartSpecTM 3000, Bio-Rad Inc., Hercules, CA, USA). The MFI and percent hemolysis were calculated as previously described by Raval et al. [19] using the following equations.

MFI calculation:

$$\frac{\left({\mathrm{fHb}}_{\mathrm{rocked}}-{\mathrm{fHb}}_{\mathrm{control}}\right)}{\left({\mathrm{Hb}}_{\mathrm{aliquot}}-{\mathrm{fHb}}_{\mathrm{control}}\right)}\times 100$$

Percent Hemolysis Equation:

$$\frac{\left(100-\mathrm{Hct}\;\mathrm{of}\;\mathrm{sample}\right)\times {\mathrm{fHb}}_{\mathrm{control}}}{{\mathrm{Hb}}_{\mathrm{aliquot}}}$$

fHb_control_ = mean free Hb concentration in the supernatant of the unrocked control sample;
fHb_rocked_ = mean free Hb concentration in the supernatant of the rocked sample;
Hb_aliquot_ = mean total Hb concentration of the RBC aliquot at an Hct of 40%.

### 2.6. Measurement of LDH Activity

LDH was measured using a Lactate Dehydrogenase Activity Assay Kit (Sigma-Aldrich, St. Louis, MO, USA) according to manufacturer’s instructions. An aliquot of RBC concentrate was frozen at −80 °C for batch analysis. In addition, an aliquot of the RBC concentrate was pelleted by centrifugation (10,000 *g*, 10 min at 4 °C) and the supernatant (plasma) stored at −80 °C. Thawed RBC samples were rapidly homogenized on ice in LDH Assay Buffer and insoluble material removed by centrifugation (10,000 *g*, 15 min at 4 °C). The soluble fraction was used for assay while plasma from RBC concentrate was assayed directly.

### 2.7. Measurement of Glutathione Content of RBC

Glutathione was measured using a Glutathione Assay Kit (Cayman Chemical, Ann Arbor, MI, USA) according to manufacturer’s instructions. An aliquot of RBC was pelleted by centrifugation (10,000 *g*, 10 min at 4 °C) and lysed in 4 volumes of cold water. After centrifugation (10,000 *g*, 15 min at 4 °C) the erythrocyte lysate was deproteinized with an equal volume of 10% metaphosphoric acid and centrifuged (10,000 *g*, 10 min at 4 °C). The supernatants were stored at −80 °C for batch analysis.

### 2.8. Western Blot for β-Spectrin

RBCs were pelleted by centrifugation (10,000 *g*, 10 min at 4 °C) and washed with cold PBS. RBCs were lysed with RIPA buffer containing protease and phosphatase inhibitors (Sigma-Aldrich, St. Louis, MO, USA). RBC lysates were resolved by SDS polyacrylamide gel electrophoresis (4–20%) and electrophoretically transferred to polyvinylidene fluoride membranes (0.2 μm pore size) according to manufacturer's instructions (Life Technologies, Carlsbad, CA, USA). Immunodetection was performed using chemiluminescence with the Renaissance Western Blot Chemiluminescence Reagent Plus (Perkin Elmer Life Sciences Inc., Boston, MA, USA). Blots were stripped using the Restore™ Western Blot Stripping Buffer (Pierce Biotechnology Inc., Rockford, IL, USA) as described by the manufacturer. Purified rabbit monoclonal antibodies to β-spectrin 1 (ab 129065, Abcam, Cambridge, MA, USA) and actin (sc-1616, Santa Cruz Biotechnology) were used in this study. Optical densities of antibody-specific bands were determined using Quantity One acquisition and analysis software (Bio-Rad, Hercules, CA, USA).

### 2.9. Scanning Electron Microscopy (SEM) of RBC

For SEM studies, RBCs were initially fixed in 0.2% glutaraldehyde in 0.1 M cacodylate buffer at room temperature for 15 min. Following centrifugation and removal of supernatant, RBCs were fixed in 1.5% glutaraldehyde in 0.1 M cacodylate buffer and stored at 4 °C before further processing at the Virginia Commonwealth University Department of Neurobiology & Anatomy Microscopy Facility. Here, the fixative was replaced with distilled water, and mounted on poly-l-lysine-coated glass slides. The glass slides were kept in a moist atmosphere and dehydrated in graded ethanol (50%, 70%, 80%, 95% and 100%). Samples were allowed to air dry, mounted on stubs with silver paint, dried overnight, followed by several coats of gold sputter, and then visualized using a Zeiss Evo 50XVP microscope (Carl Zeiss SMT, Inc., Peabody, MA, USA). RBC shapes were identified using the classification postulated by Bessis [24]. The percentages of discocytes, echinocytes, spheroechinocytes, stomatocytes, spherostomatocytes, and spherocytes were evaluated by counting 500 cells in randomly chosen fields. Reversible and irreversible shapes were determined as previously described [25]. RBC manifesting echinocyte and stomatocyte shapes are traditionally considered to be able to return to the discocyte shape under certain conditions. Thus, these RBC shape changes are considered potentially reversible transformations. In contrast, RBC assuming spheroechinocyte, spherostomatocyte, spherocyte, ovalocyte, and degenerated shapes are considered irreversibly changed cells.

### 2.10. Statistical Analysis

Statistical analysis was performed using SAS 9.4 and GraphPad Prism 7.0 (GraphPad Software, San Diego, CA, USA). Data are expressed as mean ± SE. Results were compared by one-way ANOVA and the post-hoc Tukey test to identify specific differences between groups. Statistical significance was confirmed at a *p*-value of < 0.05. 

## 3. Results

### 3.1. Intracellular VitC Concentrations in RBC

**Figure 1:**
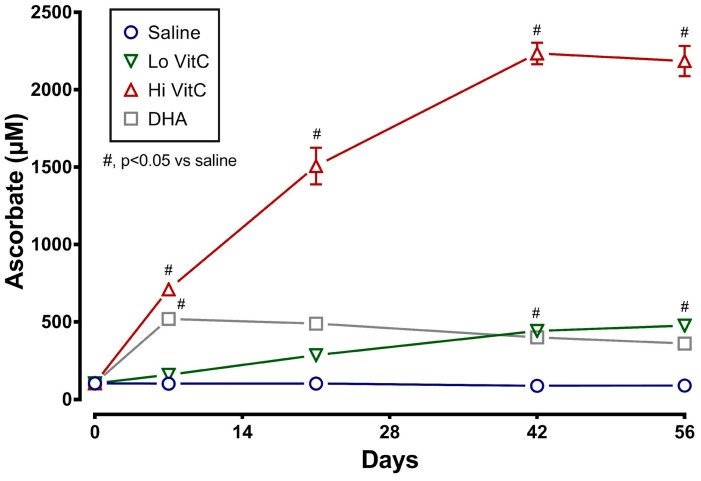
VitC exposure increased intracellular RBC ascorbate concentrations during storage. RBCs supplemented with reduced VitC (Lo/Hi) or oxidized VitC (DHA) had significantly higher intracellular levels of VitC compared to saline controls. Data are significant for Lo VitC on Days 42 and 56, for Hi VitC on Days 7, 21, 42, and 56, and for DHA on Day 7. (*N* = 5/group, *p* < 0.05).

Measurement of intracellular VitC content showed that RBC stored for 56 days at 4 °C in saline (absence of VitC) are gradually depleted of VitC. The addition of exogenous VitC resulted in a statistically significant increase in RBC VitC content (Figure 1). This was particularly evident with addition of Hi VitC (3 mmol/L, *p* < 0.05) and oxidized VitC (DHA, *p* < 0.05). Intracellular RBC VitC content continued to gradually increase with Lo (*p* < 0.05, on day 42 and 56) and Hi VitC (*p* < 0.05, all time-points), but did not change further after 7 days with DHA.

### 3.2. Changes in pH

**Figure 2:**
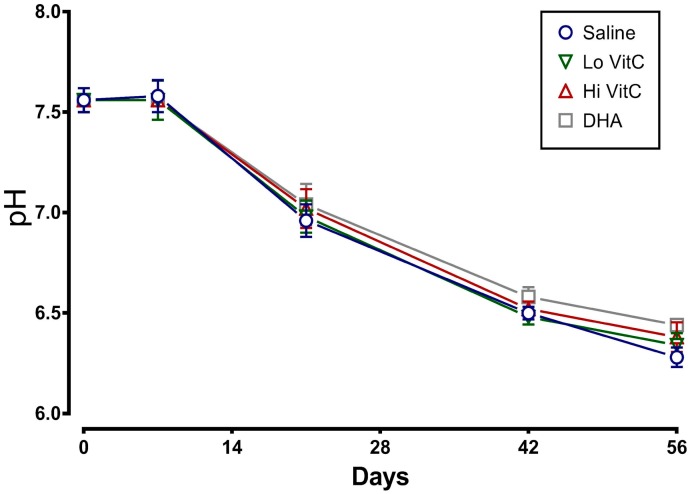
VitC exposure was associated with no changes in plasma pH. RBC concentrates showed a decline in plasma pH over 56 days of storage. The addition of reduced VitC (Lo/Hi) or oxidized VitC (DHA) did not alter the pH over the storage duration. (*N* = 5/group, *p* > 0.05).

We determined the plasma pH of packed RBC stored for 56 days in saline or VitC. As seen in Figure 2, there was a sharp decline in the pH, which started at Day 7 and continued to drop over 56 days. The addition of VitC (reduced or oxidized) had no significant impact on the decrease in pH.

### 3.3. Mean Fragility Index (MFI) and Percent Hemolysis

**Figure 3:**
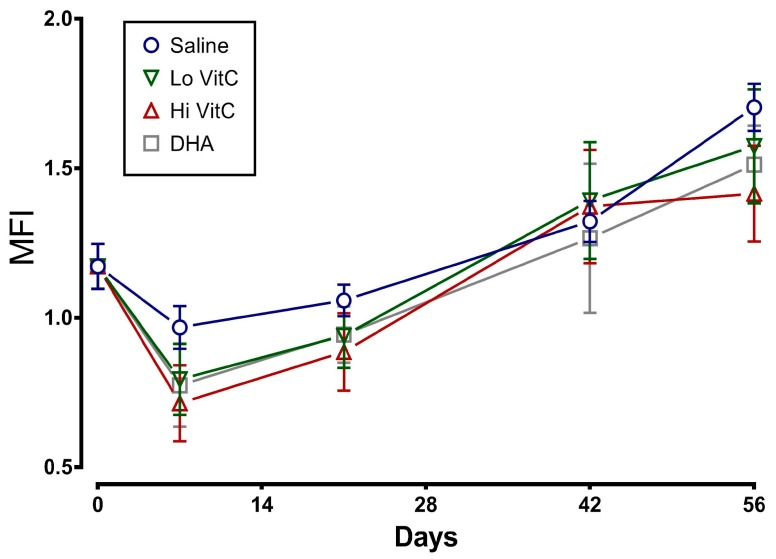
Effect of VitC exposure on the MFI of RBC. MFI of RBC treated with saline increased over 56 days of storage. The addition of reduced VitC (Lo/Hi) or oxidized VitC (DHA) reduced the MFI of treated RBC. However, this decrease did not reach statistical significance. (*N* = 5/group, *p* > 0.05).

**Figure 4:**
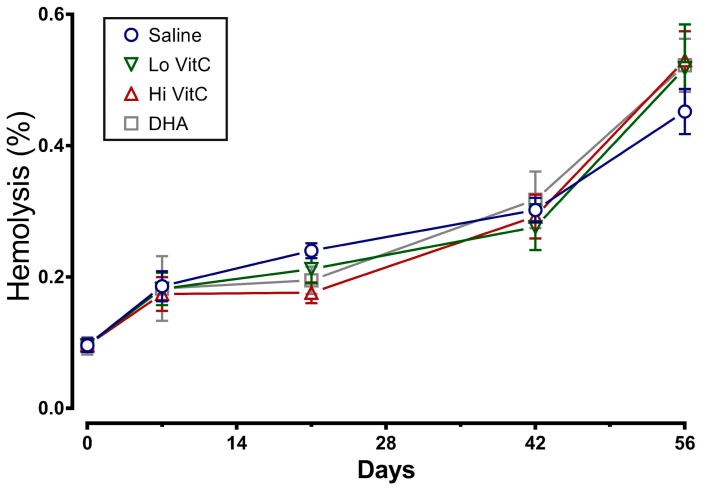
Effect of VitC exposure on percent hemolysis of RBC. The percent hemolysis of RBC treated with saline increased over 56 days of storage. The addition of reduced VitC (Lo/Hi) or oxidized VitC (DHA) did not significantly alter the percent hemolysis when compared to saline alone. (*N* = 5/group, *p* > 0.05).

To determine whether addition of VitC preserves RBC membrane integrity, we examined MFI and percent hemolysis in RBC stored ± VitC for 56 days. As seen in Figure 3, the addition of VitC reduced the MFI of treated RBC. However, the decrease did not reach statistical significance (*p* > 0.05). VitC addition also did not significantly alter the percent hemolysis when compared to saline alone (Figure 4, *p* > 0.05).

### 3.4. Lactate Dehydrogenase Activity of Stored RBC

**Figure 5:**
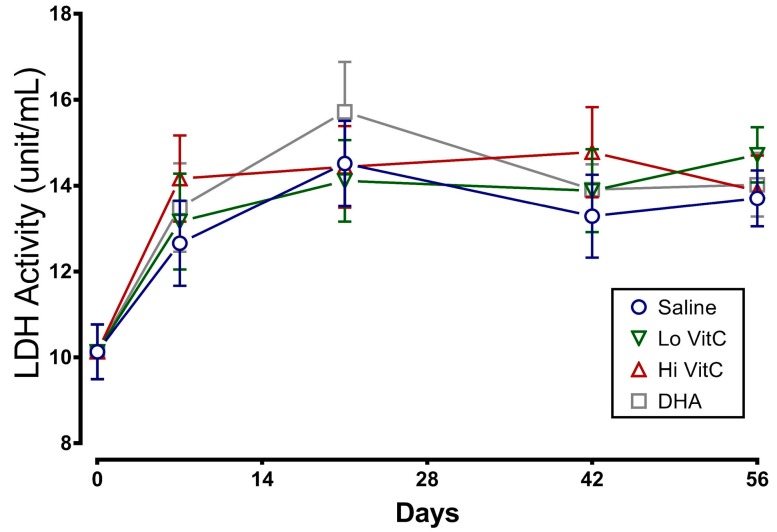
VitC exposure was associated with no changes in LDH activity. LDH activity in RBC treated with saline increased over the first 14 days and did not change further over the remaining 56 days. The addition of reduced VitC (Lo/Hi) or oxidized VitC (DHA) did not significantly alter the release of LDH from stored RBC. (*N* = 5/group, *p* > 0.05).

Another measure of membrane fragility is the release of LDH from damaged RBC. Therefore we measured LDH activity in stored RBC ± VitC for 56 days. As seen in Figure 5, LDH activity increased in the first 14 days and then plateaued out for the remaining 56 days. The addition of VitC did not significantly alter the release of LDH from stored RBC (*p* > 0.05).

### 3.5. Glutathione Content of Stored RBC

**Figure 6:**
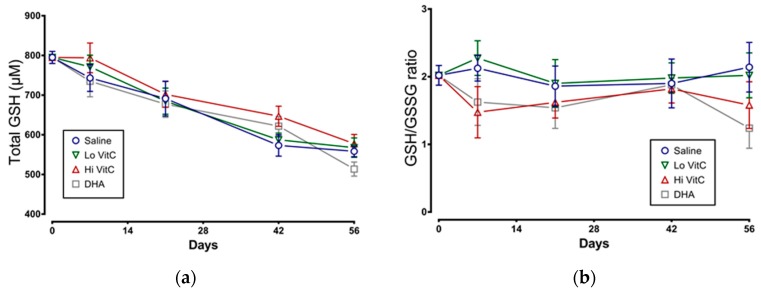
Effect of VitC exposure on glutathione content of stored RBC. (**a**) Total GSH content in RBC stored in saline decreased over 56 days. Addition of reduced VitC (Lo/Hi) or oxidized VitC (DHA) did not significantly alter the total GSH content from stored RBC. (**b**) There was a slight decrease in the GSH/GSSG ratios on Day 7 with Hi-VitC and DHA-treated RBC. However, this decrease was not statistically significant. (*N* = 5/group, *p* > 0.05).

We examined total GSH levels and the GSH/GSSG ratio in stored RBC ± VitC for 56 days. As seen in Figure 6a, total GSH content in RBC stored in saline decreased over 56 days, indicative of ongoing oxidative stress. However, the addition of VitC did not restore the GSH content in RBC. We also observed a slight decrease in the GSH/GSSG ratios on Day 7 with Hi-VitC and DHA-treated RBC (Figure 6b). However, this decrease was not statistically significant (*p* > 0.05).

### 3.6. Expression of β-Spectrin in Stored RBC

**Figure 7:**
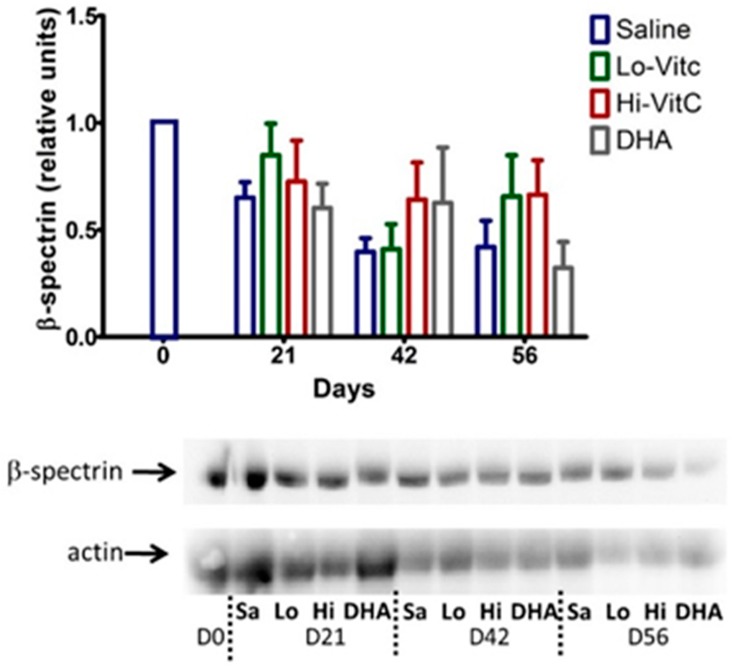
Expression of β-spectrin in stored RBC. The β-spectrin content of RBC stored in saline progressively declined over 56 days. Addition of reduced VitC (Lo/Hi) or oxidized VitC (DHA) increased β-spectrin levels of RBC over saline (Sa). However, these changes did not reach statistical significance. (*N* = 5/group, *p* > 0.05).

To determine β-spectrin levels in our study, we performed Western blot analysis of RBC ± VitC. As seen in Figure 7, β-spectrin content of RBC stored in saline progressively declined. The addition of VitC (oxidized or reduced) had a protective effect on β-spectrin levels. However, these changes did not reach statistical significance (*p* > 0.05). 

### 3.7. Electron Microscopy of Stored RBC

**Figure 8:**
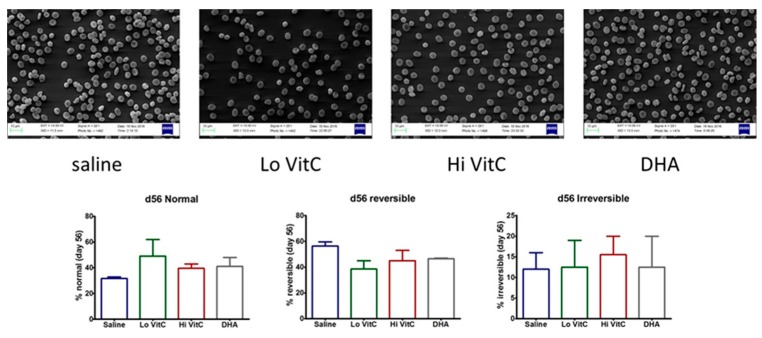
Scanning electron microscopy of RBC. RBC stored in saline undergo considerable shape change from a normal smooth discoid shape (discocyte) to a spheroid shape with speculae (echinocyte) over 56 days with only 31% exhibiting a normal discocyte appearance on Day 56. The addition of reduced VitC (Lo/Hi) or oxidized VitC (DHA) increased the percent of normal discocyte shaped RBCs on Day 56. However, these changes again did not reach statistical significance. (*N* = 3/group, *p* > 0.05).

To further examine RBC structure and integrity, we performed scanning electron microscopy (SEM) of RBC ± VitC. Stored RBCs undergo considerable shape change from a normal smooth discoid shape (discocyte) to a spheroid shape with speculae (echinocyte). While some of these changes are reversible, others are irreversible, thus making the RBC dysfunctional [15,16]. In our studies, we found that the percent of normal RBCs were higher on Day 56 in RBC stored in VitC (Figure 8). However, these changes again did not reach statistical significance (*p* > 0.05).

## 4. Discussion

Limited storage duration often results in a critical shortage of RBCs for routine/emergency use in emergency departments and critical care centers worldwide. Moreover, transfusion of these stored components to critically ill patients is often associated with increased multi-organ failure, nosocomial infections, ICU length of stay, and mortality [26,27]. Therefore, in this study, we examined the usefulness of the addition of VitC to stored RBCs. We hypothesized that VitC would reduce red blood cell storage lesions, thus prolonging their storage duration and improving their viability and function upon transfusion. While our results were promising, in that VitC addition showed beneficial trends towards better function without compromising the RBC’s internal biochemical properties, many of the aspects analyzed did not reach statistical significance due to the relatively small sample size (*n* = 5) available for the study.

Most studies that have examined the use of VitC to improve RBC storage have used the reduced form of VitC, ascorbic acid. A unique aspect of our study was the use of both reduced VitC, ascorbic acid and oxidized VitC, dehydroascorbic acid. The rationale for using DHA was that RBC have receptors only for oxidized VitC and not for reduced VitC [28,29,30]. In agreement with observation, the addition of DHA resulted in a rapid uptake of VitC by RBC (Figure 1). In contrast, the addition of the Lo VitC dose (0.3 mmol/L) only produced gradual increases in intracellular RBC ascorbic acid content. However, the addition of Hi VitC (3 mmol/L) also produced an abrupt increase in RBC ascorbic acid content, which was sustained over 56 days (Figure 1). This is plausible only if ascorbic acid is oxidized to DHA, and the DHA is subsequently taken up via the glucose transporters. On this basis we conclude that there is ongoing oxidative stress in RBC stored for 56 days at 4 °C. Moreover, addition of reduced or oxidized VitC enhances RBC anti-oxidant levels by increasing their ascorbic acid content. However, in light of recent studies by Zhang et al., in which they showed that human erythrocytes express high levels of Glut1, take up DHA, and reduce it to VitC at the expense of reducing endogenous GSH, care must be employed with the use of DHA to increase RBC anti-oxidant stores [13].

Stored RBC are subject to oxidative stress induced storage lesions. Storage lesions deplete available adenine nucleotide pool (ATP, ADP, AMP) and 2,3-bisphosphoglyceric acid (2,3-DPG) levels resulting in limited oxygen unloading from hemoglobin upon systemic perfusion. A major factor causing the decrease of 2,3-DPG during storage is the fall in the pH of RBC. However, in agreement with Dawson et al. [31] and Raval et al. [19], addition of VitC did not negatively impact the pH of RBC (Figure 2). Moreover, the biochemical parameters of RBC units stored in VitC were similar to the saline control RBCs (Figure 3, Figure 4, Figure 5 and Figure 6), demonstrating that VitC exposure did not adversely alter the biochemistry of RBCs at the dose utilized in these experiments.

Oxidative damage to RBC during storage alters cytoskeletal proteins and membrane phospholipids, resulting in increased osmotic fragility during storage and release of RBC contents into the supernatant plasma [11,12]. Others have previously shown that the MFI, a measure of RBC membrane sub-lethal injury, was found to be significantly lower in RBCs treated with 5.86 mmol/L or greater concentration of ascorbic acid by day 42 of storage [19]. Similarly, the percent hemolysis of ascorbic acid-treated RBCs was also significantly lower compared to the saline controls by Storage Day 21 when 8.78 mmol/L ascorbic acid was used [22]. In our study, using 0.3 mmol/L and 3 mmol/L VitC, we found similar beneficial trends in MFI and percent hemolysis (Figure 3 and Figure 4). However, these changes did not reach statistical significance due to the smaller sample size of our study. The reason for using lower doses of VitC in our study when compared to others was to overcome the putative pro-oxidant activity of high concentrations of VitC that have been reported by some groups [32,33] to cause oxidative injury [31,34].

Total glutathione (GSH) content and the ratio of reduced over oxidized glutathione (GSH/GSSG) is an indicator of oxidative stress in stored RBC. In this study, addition of VitC did not alter the total GSH content or the GSH/GSSG ratio (Figure 6). Some studies have shown that, under physiological conditions, once DHA is taken up by cells, it is reduced intracellularly to VitC at the expense of GSH [35] or homocysteine [36]. However, the reverse reaction of VitC reducing oxidized GSH and restoring GSH levels have not been reported. Our data support those observations and suggest that while VitC can serve independently as a reducing agent against many reactive oxygen species (ROS) including superoxide anion (O_2_^−1^), hydroxyl radical (OH•^−^), singlet oxygen (O_2_^∗^), and hypochlorous acid (HClO), it is incapable of donating electrons to oxidized GSH.

Tu et al. [37] previously showed that hyperglycemia in diabetes produced lower RBC ascorbate levels with increased RBC rigidity. Moreover, RBC β-spectrin expression correlated with its VitC concentrations [37] and that low β-spectrin levels were associated with reversible oxidative modification that affected RBC membrane integrity and structure. In agreement with Tu et al [37], we found that RBC storage over 56 days reduced β-spectrin levels (Figure 7). The addition of VitC (oxidized or reduced) potentially reversed these oxidative modifications and restored β-spectrin levels. This suggests that loading RBCs with VitC could restore membrane integrity and structure during storage. In agreement with these observations, using SEM we found that the percent of normal RBCs were higher on Day 56 in RBC stored in VitC compared to saline (Figure 8).

This study had several positives as well as limitations. The use of DHA, as well as the addition of VitC at the blood supplier facility, eliminated issues with transport that could have impacted the RBCs. A vast number of relevant assays were performed to determine change in RBC function over time. However, the biggest limitation was the sample size of the study, which severely impacted the statistical significance of the data. It is likely that increasing the sample size similar to that utilized by other groups [19,22] will yield statistically significant data. A second limitation was that generation of ROS was not measured directly, but was shown by indirect measures. Finally, it is possible that VitC alone may be insufficient to overcome the various aspects of RBC storage lesions. In line with this reasoning, Czubak et al. recently published a study where they showed that similar concentrations of VitC in combination with Trolox, a water soluble vitamin E analog, was required to reverse many RBC storage lesions [22].

## 5. Conclusions

In conclusion, these studies suggest that RBC stored for 56 days undergo oxidative modifications that alter their structure and function. VitC partially protects RBCs from these oxidative changes. Varying the dose of VitC, combining VitC with other antioxidants, and increasing the sample size used for this study is likely to provide more statistically significant observations.
